# Association of Tirzepatide Use With Clinical Outcomes After TAVR in Obese Patients: A Propensity Score–Matched Real-World Study

**DOI:** 10.1016/j.jscai.2026.105384

**Published:** 2026-04-23

**Authors:** Ibrahim Mortada, Valerie Quach, Aaron W. Lee, Nicholas Long, Grant Ebel, Ahmad Al Abdouh, Mohanad Albayyaa, Avery Love, Shareef Mansour, Khaled Chatila, Mostafa Shalaby, Thomas A. Blackwell, Hani Jneid

**Affiliations:** a Department of Cardiovascular Medicine, University of Texas Medical Branch, Galveston, Texas; b John Sealy School of Medicine, University of Texas Medical Branch, Galveston, Texas; c John P. and Kathrine G. McGovern Medical School, The University of Texas Health Science Center at Houston, Texas; d Department of Internal Medicine, University of Texas Medical Branch, Galveston, Texas

**Keywords:** obesity, metabolism, tirzepatide, transcatheter aortic valve replacement

## Abstract

**Background:**

Patients with obesity undergoing transcatheter aortic valve replacement (TAVR) remain at elevated risk for recurrent heart failure (HF) and cardiorenal events. Tirzepatide produces substantial weight loss and metabolic improvement, but its impact in post-TAVR populations is unknown.

**Methods:**

We performed a retrospective study using the TriNetX Global Collaborative Network. Adults with obesity who underwent TAVR between January 1, 2020, and January 1, 2025, were included. Patients initiating tirzepatide after TAVR were compared with patients not receiving tirzepatide. The primary outcome included HF events, while secondary outcomes were acute kidney injury (AKI), acute myocardial infarction (AMI), and ischemic stroke (IS). Fall events were prespecified as a falsification end point. Associations were evaluated using hazard ratios (HRs) with 95% CIs.

**Results:**

Propensity score matching (1:1) balanced demographic characteristics, comorbidities, medications, and laboratory characteristics, yielding 421 patients in each cohort. Over 1 year of follow-up, tirzepatide use was associated with a significantly lower hazard of HF (HR, 0.68; 95% CI, 0.56-0.83) and AKI (HR, 0.63; 95% CI, 0.43-0.93). Associations with AMI (HR, 0.81; 95% CI, 0.48-1.37) and IS (HR, 0.726; 95% CI, 0.45-1.17) were not statistically significant. The falsification end point was likewise similar between groups (HR, 0.82; 95% CI, 0.47-1.43).

**Conclusions:**

Among patients with obesity undergoing TAVR, tirzepatide use was associated with a lower hazard of HF and AKI within 1 year. No significant association was observed for AMI or IS. These findings suggest potential cardiorenal benefits of tirzepatide in high-risk structural heart populations and warrant prospective evaluation.

## Introduction

Aortic stenosis (AS) is one of the most prevalent heart valve pathologies, with approximately 17% of cases attributable to obesity.^1^ Transcatheter aortic valve replacement (TAVR) has grown to become an exceedingly useful therapy in the treatment of AS.^2^^,^^3^ Although it was initially reserved for AS cases at too high-risk for surgery, it has since grown to include the full spectrum of AS severity, replacing surgical aortic valve replacement owing to its minimal invasiveness, shorter hospital stays, and faster recovery.^4^ Prior studies have reported one-third of patients who require TAVR to be overweight and an additional one-third to be obese.^5^ Obesity is associated with adverse cardiometabolic profiles including insulin resistance, heart failure (HF), and chronic kidney disease.^6^ These profiles can influence postprocedural outcomes and contribute to an increase in all-cause mortality.^7^ Therefore, obese patients following TAVR are at a higher risk for adverse cardiovascular events, highlighting the need for targeted therapies for this population. One potential therapy is tirzepatide, a dual agonist for glucagon-like peptide (GLP)-1 and glucose-dependent insulinotropic polypeptide receptors used for type 2 diabetes. Recent studies have shown tirzepatide’s ability to produce sustainable weight loss, improve glycemic control, and control blood pressure and other cardiometabolic functions.^8^ While there is evidence suggesting a decrease in adverse cardiovascular events with tirzepatide in patients with obesity, there remains a lack in understanding how tirzepatide affects structural heart diseases, specifically following TAVR.^9^ Accordingly, we conducted a retrospective cohort study to examine the association between tirzepatide use and 1-year clinical cardiorenal outcomes in obese patients following TAVR.

## Methods

This retrospective, observational study was conducted using TriNetX, a federated research network containing deidentified electronic medical record (EMR) data aggregated from participating health care organizations. The analysis was performed using the Global Collaborative Network and included data from approximately 170 health care organizations. Data elements include patient demographic characteristic information, diagnoses and procedures, medications, laboratory values, clinical encounters, and outcomes. TriNetX allows for real-time analysis using built-in analytic tools with data refreshed regularly. Our study was conducted in accordance with the Strengthening the Reporting of Observational Studies in Epidemiology guidelines and followed TriNetX publication standards. All clinical codes used to define cohorts and outcomes are provided in a Supplemental Table S1.

### Study cohorts

The study population included adult patients aged ≥18 years who underwent TAVR with a diagnosis of obesity. Index procedures occurring between January 1, 2020, and January 1, 2025, were eligible for inclusion. Patients meeting criteria were stratified into an exposed cohort consisting of individuals with documented tirzepatide use and a comparator cohort consisting of individuals without documented tirzepatide use. Tirzepatide exposure was defined as any documented prescription following TAVR. Patients were classified into the tirzepatide cohort at the time of first prescription; however, owing to platform constraints, exposure was analyzed as a fixed variable beginning at index.

### Outcomes

Outcomes were assessed beginning 1 day after the index date through 1 year following TAVR. The primary outcome was HF. Secondary outcomes included acute kidney injury (AKI), acute myocardial infarction (AMI), and ischemic stroke (IS). A falsification end point (unspecified fall) was evaluated to assess for residual confounding. For time-to-event analyses, patients were included regardless of whether they had a documented history of the outcome prior to the index date, consistent with the analytic settings used in TriNetX. Patients were followed up until the occurrence of the outcome, death, last recorded clinical encounter, or completion of the 1-year observation window, whichever occurred first.

### Propensity matching

Matching was performed to improve comparability between exposure groups and reduce confounding; 1:1 propensity score matching (PSM) was implemented, and a logistic regression was used to estimate propensity scores with a greedy nearest-neighbor matching algorithm and a fixed caliper of 0.1 across 26 covariates. Data rows were randomized within TriNetX before matching to limit bias related to the matching sequence. Covariates included demographic characteristics (age, sex, and race/ethnicity), cardiovascular comorbidities, noncardiovascular comorbidities, and baseline medication use. Covariate variables were chosen in advance based on clinical relevance, use in existing literature, and known associations with post-TAVR outcomes, with data-driven covariate selection avoided. Covariate balance was assessed using standardized mean differences with a prespecified threshold for acceptable balance of <0.1. A full list of covariates included in the matching process are provided in Supplemental Table S1.

### Statistical analyses

All analyses were conducted within the TriNetX Compare Outcomes framework. The types of analysis used include measures of association (risk, risk difference, risk ratio, and odds ratio), time-to-event analyses (Kaplan-Meier curves, log-rank tests, and Cox proportional hazards models), and analyses for recurrent events and laboratory values. Outcomes were compared in unmatched cohorts and propensity score–matched cohorts. Statistical significance was determined using 2-sided *P* values, with a prespecified α level of ≤.05. The cohort containing patients treated with tirzepatide serves as the reference category for hazard ratios (HRs) throughout the article.

### Institutional review board statement

This study was performed using deidentified EMR data provided by TriNetX and was deemed exempt from institutional review board oversight by the University of Texas Medical Branch (UTMB). The UTMB institutional review board determined that this study involved no direct interaction with human subjects and did not allow for access to identifiable patient information. All data collected conforms to deidentification standards outlined in Section §164.514(a) of the Health Insurance Portability and Accountability Act (HIPAA) Privacy Rule.

## Results

### Cohort identification and matching

**Figure 1:**
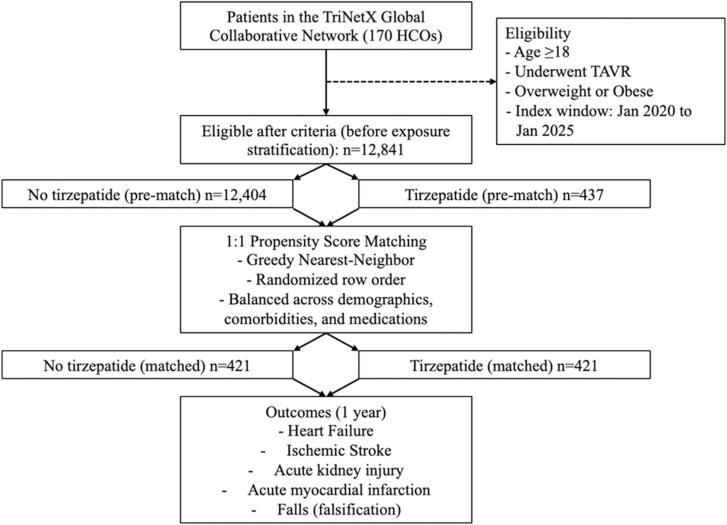
**Study flow diagram of cohort selection, propensity score matching, and outcome assessment in patients with obesity undergoing TAVR with and without tirzepatide use.** HCO, health care organization; TAVR, transcatheter aortic valve replacement.

A total of 12,404 adult patients with obesity undergoing TAVR who were not treated with tirzepatide and 437 patients treated with tirzepatide met eligibility criteria prior to PSM. After matching, 421 patients remained in each cohort for the final analytic comparison (Figure 1). Postmatching assessment demonstrated substantial improvement in balance across demographic characteristics, baseline comorbidities, and medication exposures, with most standardized mean differences below accepted thresholds, indicating adequate overall covariate balance (Table 1). Residual imbalance persisted for anticoagulant use and antilipemic therapy, although these values were markedly improved compared with prematching differences. Overall, postmatching balance was acceptable for the majority of prespecified covariates (Table 1).

**Table 1:** Baseline characteristics of patients before and after PSM

Variable	Pre-PSM tirzepatide (N = 437)	Pre-PSM control (N = 12,406)	Pre-PSM SMD	Post-PSM tirzepatide (N = 421)	Post-PSM control (N=421)	Post-PSM SMD
Demographics						
Age (mean ± SD)	72.9 ± 7.3	74.7 ± 7.7	0.243	72.9 ± 7.3	72.5 ± 7.7	0.053
White, n (%)	388 (88.8%)	10979 (88.5%)	0.01	373 (88.6%)	379 (90.0%)	0.046
Female, n (%)	214 (49.0%)	5570 (44.9%)	0.082	207 (49.2%)	214 (50.8%)	0.033
Black, n (%)	21 (4.8%)	782 (6.3%)	0.064	21 (5.0%)	18 (4.3%)	0.034
Diagnoses						
Heart failure, n (%)	242 (55.4%)	7717 (62.2%)	0.14	236 (56.1%)	251 (59.6%)	0.072
Hypertension, n (%)	387 (88.6%)	11203 (90.3%)	0.056	373 (88.6%)	377 (89.5%)	0.03
Atrial fibrillation, n (%)	150 (34.3%)	4652 (37.5%)	0.066	149 (35.4%)	137 (32.5%)	0.06
Ischemic heart disease, n (%)	281 (64.3%)	9987 (80.5%)	0.369	276 (65.6%)	286 (67.9%)	0.05
Chronic kidney disease, n (%)	163 (37.3%)	4379 (35.3%)	0.042	152 (36.1%)	170 (40.4%)	0.088
Diabetes mellitus, n (%)	307 (70.3%)	6339 (51.1%)	0.401	293 (69.6%)	308 (73.2%)	0.079
Obesity, n (%)	312 (71.4%)	7791 (62.8%)	0.185	300 (71.3%)	315 (74.8%)	0.08
Medications						
Beta blockers, n (%)	248 (56.8%)	7071 (57.0%)	0.004	240 (57.0%)	254 (60.3%)	0.068
Diuretics, n (%)	258 (59.0%)	7171 (57.8%)	0.025	253 (60.1%)	272 (64.6%)	0.093
Calcium channel blockers, n (%)	158 (36.2%)	6588 (53.1%)	0.346	152 (36.1%)	165 (39.2%)	0.064
ACE inhibitors, n (%)	87 (19.9%)	2779 (22.4%)	0.061	84 (20.0%)	95 (22.6%)	0.064
Angiotensin II inhibitors, n (%)	150 (34.3%)	3250 (26.2%)	0.178	142 (33.7%)	141 (33.5%)	0.005
Antiplatelets, n (%)	200 (45.8%)	7952 (64.1%)	0.375	197 (46.8%)	204 (48.5%)	0.033
Anticoagulants, n (%)	240 (54.9%)	9689 (78.1%)	0.506	238 (56.5%)	262 (62.2%)	0.116
Antilipemic agents, n (%)	299 (68.4%)	7989 (64.4%)	0.085	286 (67.9%)	306 (72.7%)	0.104
Antihypertensives, n (%)	86 (19.7%)	2407 (19.4%)	0.007	82 (19.5%)	91 (21.6%)	0.053
SGLT2i, n (%)	127 (29.1%)	1129 (9.1%)	0.526	115 (27.3%)	129 (30.6%)	0.073
GLP-1 analogues, n (%)	174 (39.8%)	608 (4.9%)	0.923	158 (37.5%)	163 (38.7%)	0.024
Potassium sparing diuretics, n (%)	73 (16.7%)	1439 (11.6%)	0.147	70 (16.6%)	72 (17.1%)	0.013
Anthropometric and laboratory values						
BMI (mean ± SD)	38.5 ± 7.0	34.5 ± 6.2	0.599	38.4 ± 7.1	36.9 ± 6.5	0.209
25–30 kg/m^2^, n (%)	51 (11.7%)	3261 (26.3%)	0.379	51 (12.1%)	44 (10.5%)	0.053
30–35 kg/m^2^ n (%)	172 (39.4%)	5997 (48.3%)	0.182	170 (40.4%)	167 (39.7%)	0.015
35–40 kg/m^2^ n (%)	167 (38.2%)	4250 (34.3%)	0.082	161 (38.2%)	167 (39.7%)	0.029
≥40 kg/m^2^ n (%)	171 (39.1%)	2634 (21.2%)	0.398	159 (37.8%)	153 (36.3%)	0.03
BNP (mean ± SD)	413.7 ± 1068.8	952.8 ± 3164.1	0.228	421.3 ± 1082.2	941.5 ± 4462.4	0.16
0–150, pg/mL, n (%)	45 (10.3%)	1700 (13.7%)	0.105	44 (10.5%)	49 (11.6%)	0.038
150–300, pg/mL, n (%)	20 (4.6%)	1100 (8.9%)	0.172	19 (4.5%)	20 (4.8%)	0.011
300–450, pg/mL, n (%)	12 (2.7%)	679 (5.5%)	0.138	12 (2.9%)	15 (3.6%)	0.04
450–600, pg/mL, n (%)	12 (2.7%)	464 (3.7%)	0.056	12 (2.9%)	14 (3.3%)	0.027
≥600, pg/mL, n (%)	16 (3.7%)	1305 (10.5%)	0.27	16 (3.8%)	20 (4.8%)	0.047
NT-proBNP (mean ± SD)	2483.7 ± 5773.6	3599.6 ± 7816.6	0.162	2512.8 ± 5802.0	2877.4 ± 6719.4	0.058
0–300, pg/mL, n (%)	31 (7.1%)	928 (7.5%)	0.015	30 (7.1%)	35 (8.3%)	0.045
300–600, pg/mL, n (%)	20 (4.6%)	721 (5.8%)	0.056	20 (4.8%)	22 (5.2%)	0.022
600–900, pg/mL, n (%)	11 (2.5%)	494 (4.0%)	0.083	11 (2.6%)	15 (3.6%)	0.055
900–1200, pg/mL, n (%)	10 (2.3%)	396 (3.2%)	0.055	10 (2.4%)	10 (2.4%)	<0.001
≥1200, pg/mL, n (%)	35 (8.0%)	1864 (15.0%)	0.221	35 (8.3%)	37 (8.8%)	0.017

Values are n (%) or mean ± SD.

ACE, angiotensin-converting enzyme; ARB, angiotensin II receptor blocker; BMI, body mass index; BNP, B-type natriuretic peptide; GLP-1, glucagon-like peptide-1; NT-proBNP, N-terminal pro–B-type natriuretic peptide; PSM, propensity score matching; SGLT2i, sodium-glucose co-transporter 2 inhibitor; SMD, standardized mean difference.

### Main results

**Figure 2:**
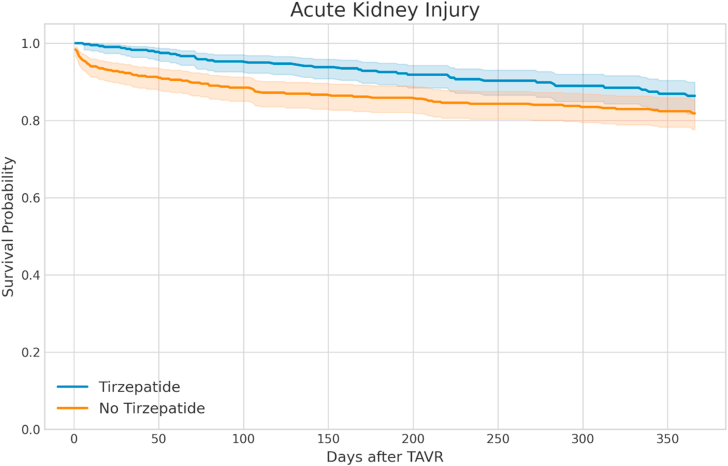
**Kaplan-Meier curve for 1-year acute kidney injury events after propensity score matching in patients with obesity undergoing TAVR with and without tirzepatide.** TAVR, transcatheter aortic valve replacement.

In the postmatching cohorts, HF occurred less frequently among patients treated with tirzepatide compared with that among those not treated with tirzepatide (44.9% vs 55.3%), corresponding to a significantly lower hazard of HF (HR, 0.68; 95% CI, 0.56-0.83) (Table 2). Kaplan-Meier analysis demonstrated early and sustained separation of the survival curves, with higher freedom from HF observed in the tirzepatide group throughout the 1-year follow-up period. Similarly, AKI occurred less frequently in the tirzepatide cohort (9.7% vs 17.1%), corresponding to a significantly lower hazard of AKI among tirzepatide-treated patients (HR, 0.63; 95% CI, 0.43-0.93). Time-to-event analysis showed consistent divergence between groups over follow-up, with a higher probability of remaining free from AKI among patients treated with tirzepatide (Figure 2). Rates of AMI were low and comparable between cohorts (5.7% vs 8.1%), with no statistically significant difference in hazard observed (HR, 0.81; 95% CI, 0.48-1.37). Rates of IS were numerically lower among patients treated with tirzepatide (6.7% vs 10.5%); however, survival analysis demonstrated no statistically significant difference in hazard between groups (HR, 0.73; 95% CI, 0.45-1.17). Consistent with falsification end point expectations, the incidence of the falsification outcome was similar between cohorts (5.0% vs 7.4%), with no statistically significant difference observed (HR, 0.82; 95% CI, 0.47-1.43), supporting the absence of substantial residual confounding.

**Table 2:** One-year clinical outcomes after propensity score matching in patients with obesity undergoing TAVR with and without tirzepatide therapy

Outcome	Tirzepatide, n/N (%)	Control, n/N (%)	ARD (95% CI)	RR (95% CI)	OR (95% CI)	HR (95% CI)	*P*
HF	189/421 (44.9)	233/421 (55.3)	−0.105 (−0.172 to −0.037)	0.81 (0.71-0.93)	0.66 (0.50-0.86)	0.68 (0.56-0.83)	.0001
AKI	41/421 (9.7)	72/421 (17.1)	−0.074 (−0.119 to −0.028)	0.57 (0.40-0.82)	0.52 (0.35-0.79)	0.63 (0.43-0.93)	.019
AMI	24/421 (5.7)	34/421 (8.1)	−0.024 (−0.058 to 0.010)	0.71 (0.43-1.17)	0.69 (0.40-1.18)	0.81 (0.48-1.37)	.433
IS	28/421 (6.7)	44/421 (10.5)	−0.038 (−0.076 to −0.000)	0.64 (0.40-1.00)	0.61 (0.37-1.00)	0.73 (0.45-1.17)	.185
Falls (falsification)	21/421 (5.0)	31/421 (7.4)	−0.024 (−0.056 to 0.009)	0.68 (0.40-1.16)	0.66 (0.37-1.17)	0.82 (0.47-1.43)	.487

AMI, acute myocardial infarction; AKI, acute kidney injury; ARD, absolute risk difference; HR, hazard ratio; IS, ischemic stroke; OR, odds ratio; RR, risk ratio; TAVR, transcatheter aortic valve replacement.

## Discussion

In this large, multicenter propensity score–matched analysis of patients with obesity undergoing TAVR, tirzepatide use was associated with significantly lower risks of HF and AKI at 1 year, while rates of myocardial infarction and IS were similar between groups. Separation of Kaplan-Meier curves occurred early and was sustained throughout follow-up, suggesting that cardiometabolic therapy may influence postprocedural trajectories soon after TAVR (Central Illustration).

**Central Illustration:**
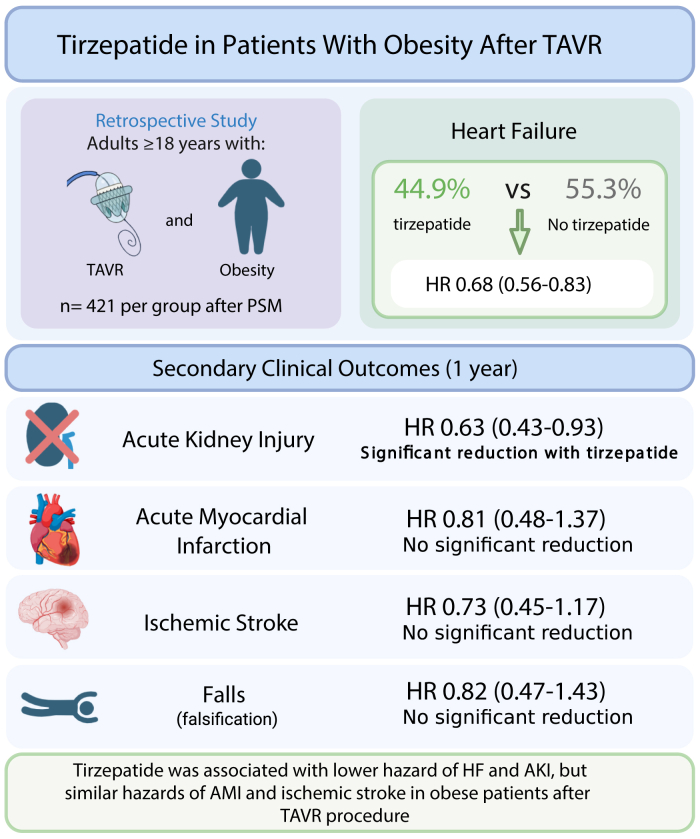
**Association of tirzepatide use with clinical outcomes after TAVR in obese patients.** HR, hazard ratio; PSM, propensity score matching; TAVR, transcatheter aortic valve replacement.

Despite successful relief of valvular obstruction with TAVR, a substantial proportion of patients remain at risk for persistent or recurrent HF driven by myocardial remodeling, diastolic dysfunction, systemic inflammation, and extracardiac metabolic stressors. Obesity is highly prevalent in contemporary TAVR populations and contributes to a cardiometabolic phenotype characterized by insulin resistance, endothelial dysfunction, and adverse ventricular–vascular coupling.^10^^,^^11^ At the same time, an obesity paradox has been described in structural heart disease, in which overweight and obese patients undergoing TAVR appear to have similar or even lower mortality compared with normal–body mass index (BMI) counterparts.^12^ In a large meta-analysis including >70,000 TAVR-treated patients, overweight and obesity were associated with lower midterm and long-term mortality but higher rates of vascular complications and pacemaker implantation, underscoring the complex and potentially bidirectional relationship between BMI and outcomes in this population.^13^ These findings suggest that BMI alone may be an imperfect surrogate for cardiometabolic health and that excess adiposity may coexist with both protective and harmful physiologic effects. Importantly, the persistence of HF and cardiorenal events after TAVR likely reflects underlying metabolic dysfunction rather than body size alone, highlighting the need for adjunctive therapies targeting cardiometabolic pathways rather than weight classification alone.^14^

The findings of the present study align with a growing body of randomized evidence demonstrating cardiovascular and HF benefits of incretin-based and metabolic therapies in patients with obesity. In the Semaglutide Effects on Cardiovascular Outcomes in People with Overweight or Obesity trial, semaglutide significantly reduced major adverse cardiovascular events among patients with obesity without diabetes, supporting the concept that weight-targeted pharmacotherapy can modify cardiovascular risk independent of glycemic control.^15^ Similarly, the Semaglutide in Patients with Heart Failure with Preserved Ejection Fraction trial demonstrated that semaglutide improved symptoms, exercise capacity, and quality of life in patients with obesity-related heart failure with preserved ejection fraction, a phenotype highly relevant to many post-TAVR patients.^16^ More recently, the Tirzepatide for Heart Failure with Preserved Ejection Fraction and Obesity trial showed that tirzepatide improved functional capacity and reduced HF events in patients with obesity-related heart failure with preserved ejection fraction, providing direct evidence for the potential of dual glucose-dependent insulinotropic polypeptide/GLP-1 agonism to influence HF trajectories in metabolically driven disease.^9^

Mechanistically, tirzepatide may reduce HF risk through several interrelated pathways. Substantial weight loss and improved glycemic control are associated with reductions in left ventricular filling pressures, systemic inflammation, and neurohormonal activation.^17^^,^^18^

Tirzepatide-based therapies have also been shown to improve endothelial function, reduce epicardial adiposity, and favorably influence myocardial energetics. These effects may attenuate the persistent hemodynamic and inflammatory stress that remains even after correction of valvular obstruction.^19^^,^^20^ In addition, improvements in blood pressure, insulin resistance, and systemic inflammation may contribute to renal protection, which is consistent with the lower hazard of AKI observed in this study.^21^, ^22^, ^23^

Emerging evidence in structural heart populations further supports the potential role of metabolic therapy after TAVR. In the Dapagliflozin in Patients Undergoing Transcatheter aortic valve replacement trial, initiation of dapagliflozin following TAVR reduced the composite of death and worsening HF, suggesting that targeted cardiometabolic therapy can improve outcomes in this high-risk population.^24^ While sodium-glucose cotransporter 2 (SGLT2) inhibitors and tirzepatide act through distinct mechanisms, both target the metabolic milieu that contributes to persistent postprocedural risk. The convergence of these findings across therapeutic classes supports the concept that structural heart interventions and metabolic optimization may be complementary rather than independent strategies.

Notably, no significant differences were observed in AMI or IS. This suggests that the observed benefits may be more specific to HF and renal pathways rather than reflecting a broad reduction in atherosclerotic events. A neutral falsification end point further reduces the likelihood that the findings are solely attributable to systematic residual confounding. Prior meta-analyses of GLP-1 receptor agonist cardiovascular outcome trials have demonstrated consistent reductions in major cardiovascular events, particularly driven by stroke and atherosclerotic end points, but have shown more variable effects on HF outcomes.^25^ The present analysis extends these observations by suggesting a potential role for dual incretin-based therapy in reducing HF-related risk in structurally complex populations such as TAVR recipients.

### Limitations

This study has several important limitations. As a retrospective observational analysis using EMR data, causal inference cannot be established and residual confounding may persist despite extensive PSM across demographic characteristics, comorbidities, medications, and laboratory variables. In particular, exposure classification may introduce immortal time bias and time-dependent misclassification. Patients in the tirzepatide cohort were required to survive long enough after TAVR to initiate therapy, and HF events that occurred in the interval between the TAVR procedure and the start of tirzepatide would not be attributed to the exposed group. Consequently, early postprocedural HF events may have been preferentially captured in the comparator cohort, potentially exaggerating the apparent reduction in HF risk associated with tirzepatide. This form of bias is well described in pharmacoepidemiologic studies when treatment exposure is defined after cohort entry and cannot be modeled as a time-varying variable.^26^, ^27^, ^28^ Time-dependent exposure modeling and landmark analyses were not feasible within the TriNetX framework and may influence the magnitude of the observed associations.

Granular clinical and physiologic data were also unavailable, including echocardiographic measures such as diastolic function indices, left ventricular mass, prosthetic valve gradients, and pulmonary pressures, all of which are important determinants of post-TAVR HF risk. We were unable to assess the magnitude or trajectory of weight loss, glycemic response, and duration of therapy, exercise, or medication adherence, which limits mechanistic interpretation. TriNetX privacy policies preclude assessment of clustering by site, hospital volume, or geographic region, preventing evaluation of institutional variation in post-TAVR care pathways. Finally, reliance on diagnostic and procedural coding may introduce misclassification of exposures and outcomes, although the use of a falsification end point helps mitigate concerns regarding systematic residual confounding.^26^

## Conclusions

In patients with obesity undergoing TAVR, tirzepatide use was associated with lower risks of HF and AKI at 1 year, while other major cardiovascular outcomes were similar between groups. Taken together, these findings support the hypothesis that metabolic optimization may represent an important adjunctive strategy in the care of patients with obesity undergoing TAVR. As TAVR expands into younger and lower-risk populations with a high prevalence of metabolic disease, the role of adjunctive cardiometabolic therapies warrants further investigation. Prospective randomized trials are needed to determine whether initiation of incretin-based therapies after TAVR can causally reduce HF events, define optimal timing of therapy initiation, and evaluate long-term effects on cardiac remodeling and clinical outcomes.

## CRediT authorship contribution statement

**Ibrahim Mortada:** Conceptualization, Formal analysis, Investigation, Methodology, Writing – original draft, Writing – review & editing. **Valerie Quach:** Formal analysis, Project administration, Writing – original draft. **Aaron W. Lee:** Investigation, Software, Writing – original draft, Writing – review & editing, Visualization. **Nicholas Long:** Project administration, Writing – original draft. **Grant Ebel:** Project administration, Writing – original draft. **Ahmad Al Abdouh:** Project administration, Writing – original draft. **Mohanad Albayyaa:** Project administration, Writing – review & editing. **Avery Love:** Project administration. **Shareef Mansour:** Investigation, Writing – review & editing. **Khaled Chatila:** Investigation, Writing – review & editing. **Mostafa Shalaby:** Investigation, Supervision, Writing – review & editing. **Thomas A. Blackwell:** Funding acquisition, Supervision, Validation, Writing – review & editing. **Hani Jneid:** Funding acquisition, Resources, Supervision, Writing – review & editing.

## Declaration of competing interest

The authors declared no potential conflicts of interest with respect to the research, authorship, and/or publication of this article.

## Footnotes

To access the supplementary material accompanying this article, visit the online version of the *Journal of the Society for Cardiovascular Angiography & Interventions* at 10.1016/j.jscai.2026.105384.
